# Life cycle assessment and Monte Carlo simulation to evaluate the environmental impact of promoting LNG vehicles

**DOI:** 10.1016/j.mex.2020.101046

**Published:** 2020-08-27

**Authors:** Shouheng Sun, Myriam Ertz

**Affiliations:** LaboNFC, Université du Québec à Chicoutimi, 555 Boulevard de l'Université, Chicoutimi, QC G7H 2B1, Canada

**Keywords:** Life cycle assessment, Climate change, Uncertainty analysis, Monte-Carlo simulation, Risk assessment

## Abstract

• As a novel and alternative type of fuel for heavy-duty trucks, it is very important to assess a broad array of environmental impacts of liquefied natural gas (LNG). However, few studies have evaluated comprehensively the environmental impact of LNG as an alternative fuel on human health, ecosystems and resources from a life cycle perspective. In particular, the environmental benefit of promoting LNG vehicles is often complicated and uncertain due to many variable factors, which are also often not given enough attention. This method article describes the use of a combination of life cycle assessment (LCA) and Monte Carlo simulation to evaluate the potential environmental benefits of promoting LNG heavy-duty diesel vehicles in Saguenay, a city in Canada. It not only conducts a full-range analysis of environmental impacts, but also considers the impact of joint changes in uncertain factors such as methane emission rates, energy efficiency of engine and the project promotion prospects on the environmental benefits of LNG, making life cycle environmental impact assessment more systematic and comprehensive. The paper provides the details of all the steps used in the method and can be replicated and applied to other similar studies and research settings.
• This combined approach provides a comprehensive assessment of the environmental impacts incurred by the promotion of LNG vehicles. Besides, it also provides a certain degree of risk assessment for LNG projects.
• This method takes into account the complexity of the joint change of multiple uncertainties, which makes up for the deficiencies of previous studies that only analyze one uncertainty in isolation.
• This method takes the development prospect of LNG promoting project as an uncertain factor for environmental benefit assessment.

## Graphical abstract

**Figure:**
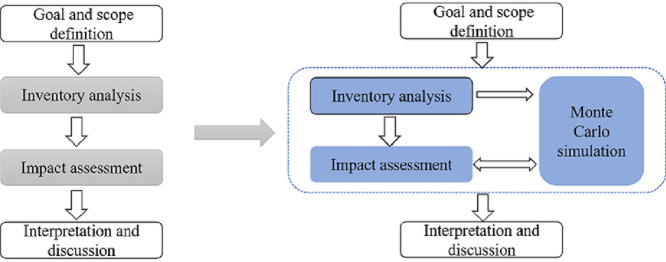

Specifications table

**Table:** 

Subject Area:	Energy
More specific subject area:	Alternative fuel vehicles, Transportation, Sustainability and the Environment
Method name:	Monte-Carlo LCA
Name and reference of original method:	LCA [1] Monte-Carlo method [2]
Resource availability:	There are no special resources. The original methods mentioned above can be used to reproduce the method.

## Backgrounds

Heavy-duty vehicles (HDVs) have gradually become the main sources of fuel consumption and emissions for the road transport sector. Since LNG can significantly reduce harmful air pollutants emitted by vehicle exhaust, it can be considered as a promising alternative fuel for HDVs. In order to better promote the sustainability of the transportation sector, it is thus important to conduct a comprehensive and systematic environmental impact assessment of LNG as an alternative fuel. However, few studies have evaluated comprehensively the environmental impact of LNG as an alternative fuel on human health, ecosystems and resources from a life cycle perspective. In particular, due to many variable factors, the environmental benefits of promoting LNG vehicles are usually complex and uncertain, which also brings great challenges to traditional LCA methodology for assessing environmental impacts.

As a methodology for assessing environmental impacts associated with all the stages of the life-cycle of a commercial product, process, or service, LCA has matured in theory and has been widely used in various fields. It addresses the potential environmental impacts (e.g. use of resources and environmental consequences of releases) throughout a product's life cycle from raw material acquisition through production, use, end-of-life treatment, recycling and final disposal (i.e. cradle-to-grave). According to the norms ISO 14040/14044, the four phases of an LCA study are as follows:

(1) goal and scope definition;
(2) inventory analysis;
(3) impact assessment;
(4) Interpretation and discussion of results.

The goal and scope definition phase, refers to the determination of the object and purpose of the LCA study and the corresponding system boundaries. Second, the inventory analysis phase involves the collection of the data necessary to meet the goals of the defined study. It is an inventory of input/output data with regard to the system being studied. The purpose of the impact assessment phase is to transform the long list of inventory data into a limited number of indicator scores by using a specific life cycle impact assessment method. These indicator scores express the relative severity on an environmental impact category, so as to better understand the environmental significance of the product, process or service under study. In the phase of interpretation and discussion, the results of the impact assessment are summarized and discussed as a basis for conclusions, recommendations and decision-making in accordance with the goal and scope definition.

However, LCA method has its own limitations in specific applications, that is, the uncertainty problem widely exists in the process of an LCA study. Among them, the uncertainty in the inventory data is the most prominent. Due to the lack of effective valid standard inventory data and the various unavoidable errors in the data collection process, the inventory data of a certain process or stage in the LCA often cannot reflect the actual situation. In many cases, the actual value of some parameters in the inventory fluctuates greatly, rather than being fixed. These uncertainties will directly affect the correctness and reliability of the LCA research conclusions.

The Monte Carlo method, also known as statistical simulation method, is a very precise method of numerical calculation guided by probability statistical theory. It uses random numbers (or more commonly pseudo-random numbers) to solve many calculation problems [2]. The Monte Carlo method is widely used in the fields of finance, macroeconomics, computational physics, and risk assessment of engineering projects]. The application of Monte Carlo method in solving practical problems has two main parts: one is to generate random variables with a certain probability distribution; the other is to estimate the numerical characteristics of the model with statistical methods, so as to obtain the numerical solution of the actual problems [2].

Monte Carlo method can randomly sample the values of uncertain variables based on probabilistic analysis, and combine with the pre-determined impact assessment method to simulate, so as to obtain statistically significant environmental impact evaluation results, which can reflect the influence of uncertain factors more accurately. Taking into account the characteristics of these two methods, we combined the Monte Carlo method and LCA and named it Monte Carlo LCA. Monte Carlo LCA method can effectively solve the uncertainty problem of LCA method in environmental impact assessment, so as to provide a more scientific and reasonable basis for decision-making.

The method thus first follows the 4-phase framework of LCA study:

(1) Goal and scope definition
(2) Inventory analysis and data collection. This step deals with data collection. Based on statistical analysis of data, it also provides uncertain parameters and corresponding distributions for the Monte Carlo simulation.
(3) Impact assessment. The statistically significant environmental impact result is calculated by combining a specific impact assessment method and Monte Carlo simulation.
(4) Interpretation and discussion of the results

Here, we take the environmental impact assessment for the project of promoting LNG HDVs in Saguenay, Canada as an example to demonstrate the use of the Monte Carlo method.

## Goal and scope definition

**Fig. 1:**
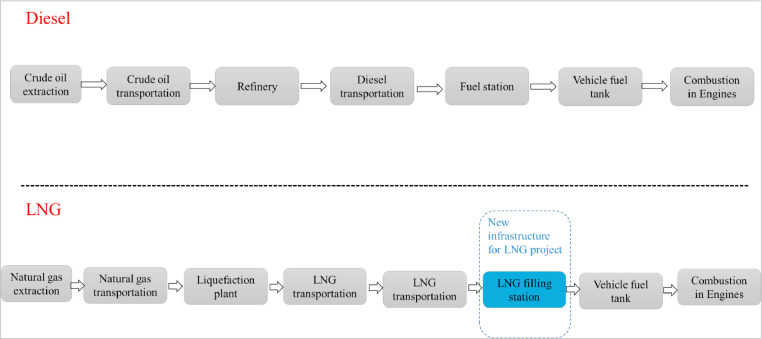
Life cycle system boundaries of Diesel and LNG.

This LCA study aims at evaluating the potential environmental benefits of deploying LNG as an alternative fuel for heavy-duty vehicles in Saguenay, Canada, instead of diesel. The research object is the life cycle of diesel and LN. The life cycle assessment (LCA) for fuels, which is known as well-to-wheels (WTW) analysis, includes measurement of energy consumption and emissions throughout the entire process of fuel production, storage, transportation and distribution (well-to-tank), and combustion in the vehicles’ fuel tank (tank-to-wheel) [3]. The infrastructures related to fuel production, transportation, storage and distribution are also included in the system boundaries. However, vehicles in the end-use phase are not included. System boundaries are shown in Fig. 1.

## Inventory analysis and data collection

Inventory analysis and data collection are based on the system boundaries. The data in this study is mainly from the Ecoinvent 3.5 database (the world's leading life cycle inventory database) [4], open data of Saguenay, and published articles.

Production and transportation of fuel. The main data collection for the production and transportation of fuel is shown in Table 1.

**Table 1:** Data collection for the production and transportation of fuel.

Process	Description and Assumption
Production and transportation of Diesel	Quebec receives crude oil from Western Canada and the U.S. Midwest through pipelines and railways. The majority of the diesel consumed in Quebec is refined in two large refineries: Montreal Refinery (Suncor) in Montreal and Jean Gaulin Refinery (Valero) in Lévis, near Quebec City. The crude oil transportation distance is estimated to be 3800 kms. The average distribution distance of diesel from refineries to fuel station is estimated at 250 km [6] . The inventory data for diesel production comes from Ecoinvent 3.5 database: “Diesel, low-sulfur {RoW}| production | APOS, U”.
Production and transportation of LNG	The natural gas used in Quebec is also transported from western Canada through pipelines. It will be liquefied at an LNG plant near Saguenay. This LNG facility will be powered by hydroelectricity from the pre-existing Saguenay grid [7]. The Ecoinvent dataset “Natural gas, high pressure {CA-QC}| natural gas, high pressure, import from CA-AB | APOS, U” is used for modeling. Gas burned for transportation, gas losses and emissions (losses and vented gas) are included. An average distance of 4000 km is estimated for the whole process of transporting. The inventory data for LNG production comes from Ecoinvent 3.5 database.

Vehicle features and fuel combustion. In North America in general, and Canada in particular, HDVs are a broad class of vehicles weighing more than 4500 kg. The data for bus and other HDVs are shown in Table 2. In addition, the tailpipe emissions data for LNG and Diesel vehicles are based on the Ecoinvent database which uses the Euro V standards. As to the methane emissions, methane emissions from the fuel tank, engine and tailpipe account for the majority. The fluctuation range is about 0.4%−1.2% [3,^8^,9]. It includes the emissions from dynamic vent, vehicle manual vent, engine crankcase and engine tailpipe. In terms of energy efficiency of LNG engine, LNG-fueled engines are less energy-efficient than modern diesel-fueled compression ignition (CI) engines [10]. LNG vehicles need more heat energy for the same traveled distance. In order to reduce the differences caused by engine technology, spark ignition engine (dedicated fuel, fueled by 100% LNG) is considered for LNG vehicles. Due to the difference between the actual use of the vehicle and the performance of the engine, the increase rate of energy consumption remains uncertain within a 10%−20% fluctuation range [8,^10^,11]. The methane emission rate and the energy efficiency of LNG engines are currently widely regarded as the main uncertain factors affecting the environmental benefits of LNG.

**Table 2:** Characteristics of Heavy-duty vehicles in Saguenay [14,15].

	Number of vehicles	Average annual distance travelled (thousands of km)	Average Fuel efficiency(L/100 km)	Total annual fuel consumption(L)
Buses	86	57.33	54.07	2,665,659
Other Heavy-duty vehicles	1665	39.40	30.90	20,270,709

Construction and operation of new filling stations. The promotion of LNG requires supporting facilities, the most important is LNG filling station. However, the actual promotion effect will be restricted by many factors, such as social and economic factors [11,13]. Since the construction and operation of filling stations have a certain impact on the environment, if the actual promotion effect is poor and the infrastructure utilization rate is low, it may have a negative impact on the overall environmental benefits of the LNG project. Therefore, the utilization rate of filling stations will bring some uncertainty to the environmental benefits of LNG. A standard small and medium-sized LNG filling station covers an area of 2000 m^2^ and is equipped with an LNG storage tank of 30 m^3^ [11,13]. The Ecoinvent database “Natural gas service station {RoW}| construction | APOS, U” is used for modeling. It includes land use for a station as well as steel requirements for compressors and containers. It is assumed that steel and concrete are recycled. A life span of 30 years is assumed. According to the characteristics of Heavy-duty vehicles in Saguenay (see Table 1) and the development scenario setting of promoting LNG (see Table 3), the fluctuation range of utilization rate is set as 30%−86%.

**Table 3:** Scenario setting for LNG HDVs promotion in Saguenay.

	Baseline	S1	S2	S3	S4	S5	S6
LNG usage scenarios	No LNG vehicles	only Bus	Bus + 5% other HDVs	Bus + 10% other HDVs	Bus+ 30% other HDVs	Bus + 50% other HDVs	Bus+ 100% other HDVs
Average daily LNG consumption (m^3^)	0	13.18–14.82	18.18- 20.46	23.19–26.10	43.23–48.64	63.27–71.19	113.37–127.55
Number of LNG filling stations	—	1	1	1	2	3	5
Capacity of LNG filling stations (m^3^)	—	30	30	30	60	90	150
Average utilization rate of Filling stations	—	44%−49%	60%−68%	77%−86%	72%−81%	70%−79%	75%−85%

Uncertain factors. Through inventory analysis and data collection, the three main uncertain factors affecting the environmental impact of LNG promotion projects and their distribution characteristics are shown in Table 4.

**Table 4:** The distribution characteristics of the uncertain variables [3,[8], [9], [10], [11], [12].

	Min	Average	Max	The standard deviation
Utilization rate of filling station	30%	58%	86%	0.0933
The increase rate of energy consumption of LNG engine to diesel engine	10%	15%	20%	0.0167
Methane emission rate	0.4%	0.8%	1.2%	0.0013

## Impact assessment

In this phase, LCA modeling and calculation were done by the SimaPro 9.0 software and the ReCiPe 2016 hierarchist impact assessment method was used for the calculation of impact indicators score related to the fuel life cycle [5]. Monte-Carlo simulation is conducted with the assistance of the Oracle Crystal Ball software. The ReCiPe 2016 hierarchist impact assessment method provides 18 midpoint indicators: Global warming, Stratospheric ozone depletion, Ionizing radiation, Ozone formation (Human health), Fine particulate matter formation, Ozone formation (Terrestrial ecosystems), Terrestrial acidification, Freshwater eutrophication, Marine eutrophication, Terrestrial ecotoxicity, Freshwater ecotoxicity, Marine ecotoxicity, Human carcinogenic toxicity, Human non-carcinogenic toxicity, Land use, Mineral resource scarcity, Fossil resource scarcity, Water consumption. These indicator scores express the relative severity on an environmental impact category [5].

The functional unit of 1 km distance travelled by vehicle was used in order to assess and compare environmental impacts. The environmental benefit of LNG can be calculated by Eq. (1).

(1) $$EB_{i}=\;\frac{EI\_LNG_{i}-EI\_Diesel_{i}}{EI-Diesel_{i}}\times 100\%$$

Among them, *i* referes to the environmental impact category, *EB_i_* refers to the environmental benefit of LNG on the environment impact category i. $\;EI\_LNG_{i}$ refers to the quantified value of environmental impact category *i* generated by an LNG vehicle traveling 1 km. $EI\_Diesel_{i}$ refers to the quantified value of the environmental impact category *i* brought by the vehicle driving with diesel for 1 km. An EB value inferior than 0 means that LNG has a lower environmental impact than diesel.

First, we use the LCA model to calculate the life cycle environmental impact of per kilogram LNG without considering uncertain factors. That is, assuming that the methane emission rate is 0, the fuel efficiency of the LNG engine is the same as that of the diesel engine, and the new LNG filling station within the system boundary of the LNG (see Fig. 1) is removed. The results are shown in Table 5.

**Table 5:** Life cycle environmental impact of per kilogram of diesel and LNG (for LNG, without considering uncertain factors).

Impact category	Unit	Diesel (per kg)	LNG (per kg)	Per LNG filling Station
Global warming	kg CO2 eq	4.08E+00	3.54E+00	6.92E+04
Stratospheric ozone depletion	kg CFC11 eq	2.11E-06	1.49E-06	3.78E-02
Ionizing radiation	kBq Co-60 eq	5.16E-02	7.45E-03	3.38E+03
Ozone formation, Human health	kg NOx eq	5.49E-03	3.53E-03	1.67E+02
Fine particulate matter formation	kg PM2.5 eq	2.34E-03	1.53E-03	1.16E+02
Ozone formation, Terrestrial ecosystems	kg NOx eq	5.79E-03	3.77E-03	1.74E+02
Terrestrial acidification	kg SO2 eq	6.32E-03	5.06E-03	2.35E+02
Freshwater eutrophication	kg P eq	1.16E-04	8.01E-05	2.73E+01
Marine eutrophication	kg N eq	1.19E-05	5.49E-06	1.51E+00
Terrestrial ecotoxicity	kg 1,4-DCB	3.88E+00	2.66E-01	2.58E+05
Freshwater ecotoxicity	kg 1,4-DCB	1.04E-02	5.30E-03	3.05E+03
Marine ecotoxicity	kg 1,4-DCB	1.76E-02	7.42E-03	4.29E+03
Human carcinogenic toxicity	kg 1,4-DCB	2.88E-02	1.92E-02	1.65E+04
Human non-carcinogenic toxicity	kg 1,4-DCB	3.16E-01	1.24E-01	8.16E+04
Land use	m2a crop eq	1.47E-02	4.25E-03	4.76E+03
Mineral resource scarcity	kg Cu eq	1.92E-03	1.46E-03	1.81E+03
Fossil resource scarcity	kg oil eq	2.63E+00	1.29E+00	1.25E+04
Water consumption	m3	2.43E-02	2.14E-02	8.12E+03

Monte-Carlo experiment can obtain and display a collection of simulation outputs for a stochastic model or for a model with stochastically varied parameters. The parameters are shown in Table 4, and the predictive function is shown in Eq. (2).

(2) $$\begin{array}{cc} EI\_LNG_{i} & =(LNG\_LCA_{i}+\alpha \times Methane\;emission\;rate+LNG\;filling\;Station\_LCA_{i}/(Utilization\;rate\;\\ & of\;filling\;station\times Capacity\;))\times ((Energy\;consumption\;of\;diesel\;vehicles\_per\;kilometer\times \\ & \left(1+increase\;rate\;of\;energy\;consumption\;of\;LNG\;engine\;to\;diesel\;engine\right))/Heat\;value\;of\;LNG\;)) \end{array}$$

Among them,$\;LNG\_LCA_{i}$ refers to the impact of each kilogram of LNG on the environmental indicator *i*, without considering the uncertain factors. *α* refers to the global warming potential of methane, which is 30 times that of CO2 in a 100-year horizon in this study. $\;LNG\;filling\;Station\_LCA_{i}$ refers to the impact of LNG filling stations on the environmental indicator *i* throughout the life cycle. *Capacity* refers to the capacity of LNG filling stations throughout the life cycle.  *Heat* *value* *of* *LNG* refers to the heat value of per kilogram of LNG, which was set to 50.4 MJ/kg in this study. Energyconsumptionofdieselvehicles_perkilometer refers to the energy required for a diesel vehicle to travel one kilometer. It can be obtained by multiplying the average Fuel efficiency of the diesel vehicles (see Table 1) and the heat value of the diesel (45.4 MJ/kg in this study).

**Fig. 2:**
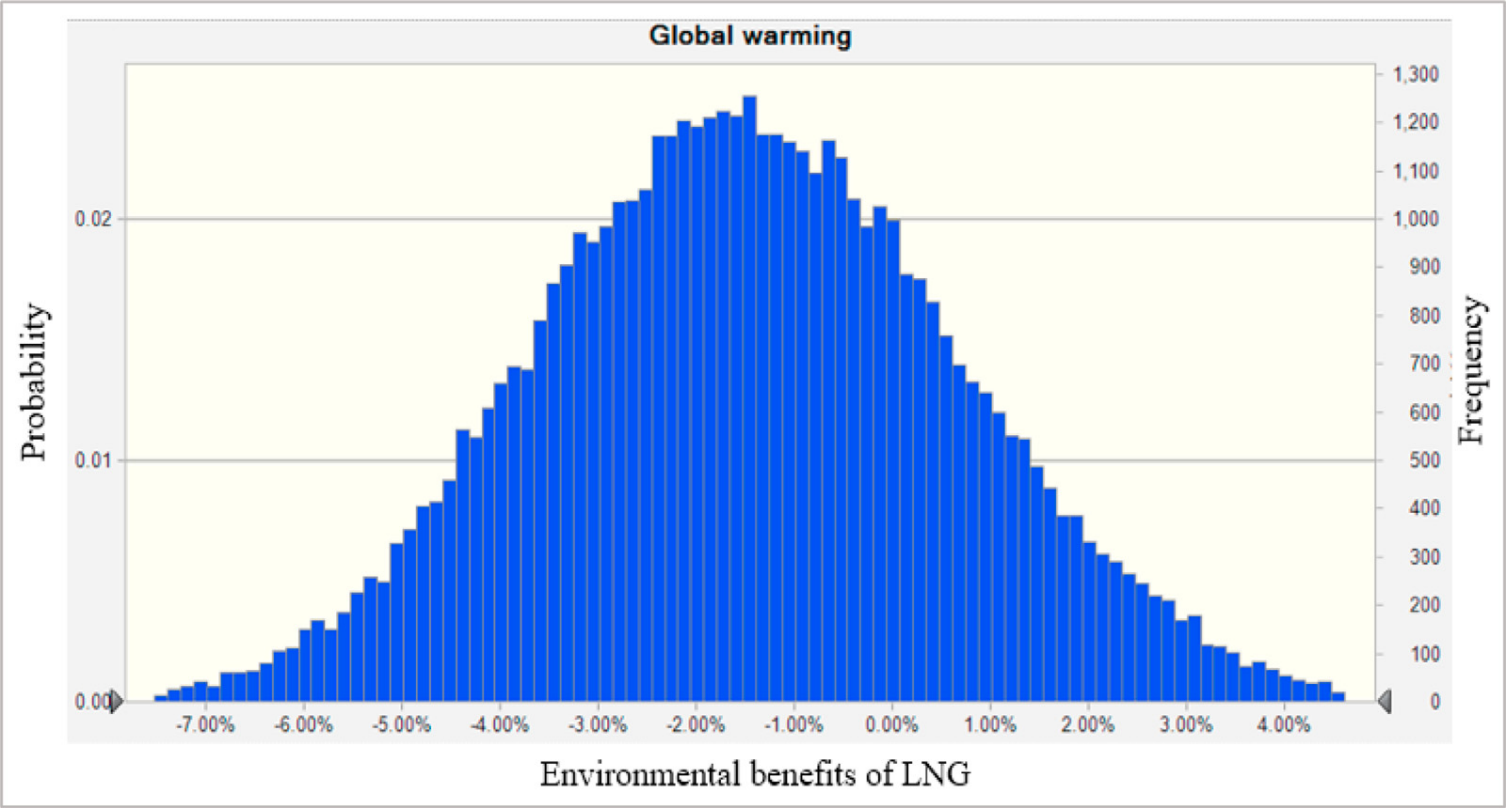
The Global warming benefits of LNG vehicles relative to diesel vehicles per kilometer distance. Note: The value of environmental benefits less than 0 means that LNG has a lower impact than diesel on the corresponding indicator.

**Fig. 3:**
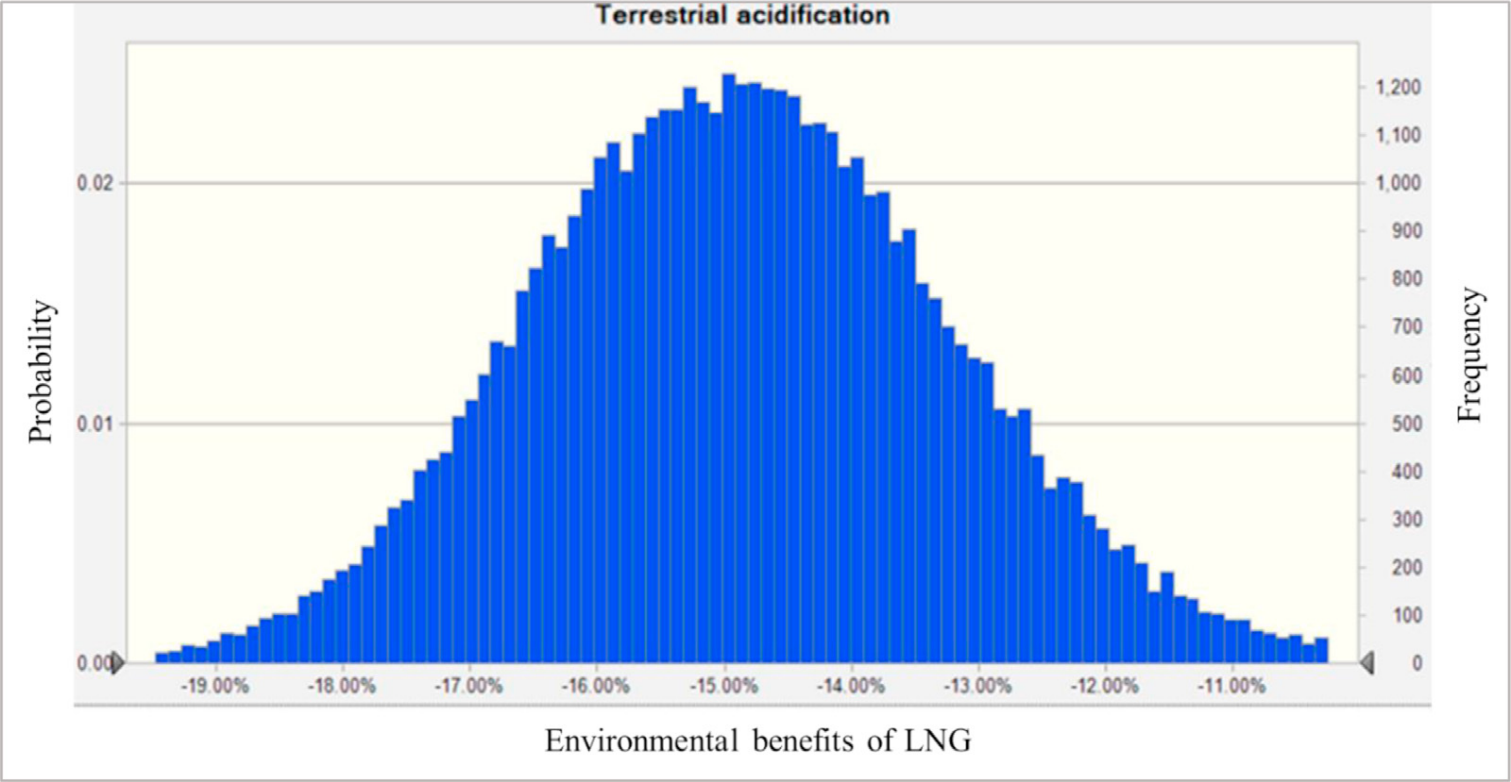
The Environmental benefits of LNG vehicles on terrestrial acidification. Note: The value of environmental benefits less than 0 means that LNG has a lower impact than diesel on the corresponding indicator.

**Fig. 4:**
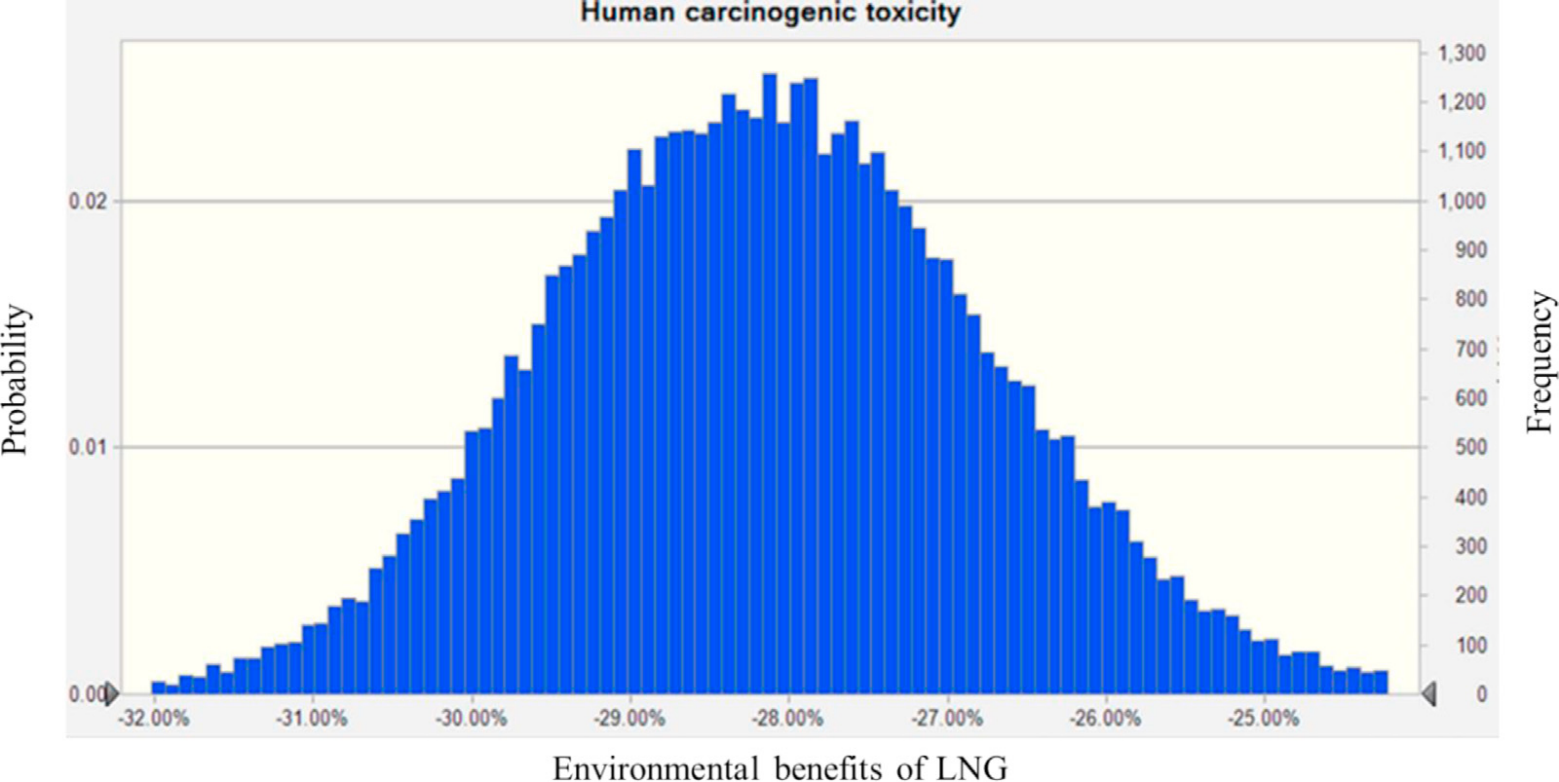
The Environmental benefits of LNG vehicles on Human carcinogenic toxicity. Note: The value of environmental benefits less than 0 means that LNG has a lower impact than diesel on the corresponding indicator.

Combined with the Eqs. (1) and (2), life cycle environmental benefit of LNG as an alternative fuel for heavy duty diesel vehicles in Saguenay is calculated based on 50,000 Monte Carlo simulations. Some of the results are shown in Figs. 2–Fig. 3, Fig. 4.

## Interpretation and discussion of the results

**Fig. 5:**
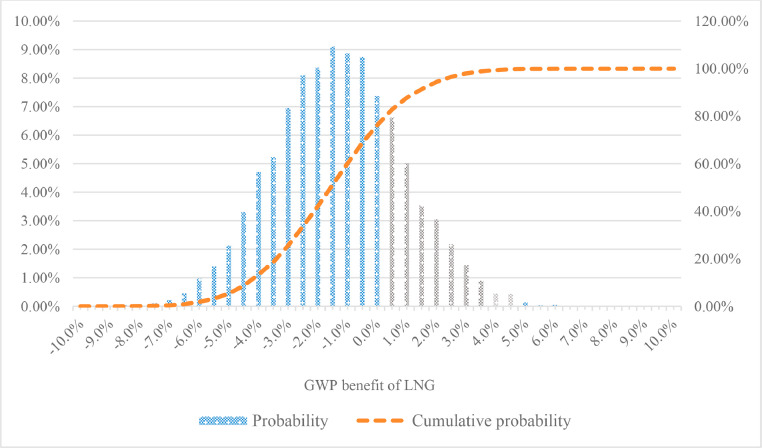
The GWP benefits distribution of LNG based on Monte Carlo simulation. Note: GWP benefit of LNG less than 0 means that LNG has lower GWP impact than diesel.

Take the environment impact indicator of global warming as the example, the potential GWP benefits distribution of LNG is shown in Fig. 5.

It can be seen from Fig. 5 that the GWP benefits of LNG follow a normal distribution with a mean value of −1.55% and a standard deviation of 2.18%. The cumulative probability that the LNG GWP benefit is less than 0 is about 76.2%. That is to say, the probability that LNG has a lower GWP impact than diesel is 76.2%.

This work provides the details of all the steps of the Monte-Carlo LCA method, including data collection. Based on these, this method can be replicated and applied to other similar studies.

## Declaration of Competing Interest

The authors declare that they have no known competing financial interests or personal relationships that could have appeared to influence the work reported in this paper.
